# A Split Forehead Flap for the Treatment of Resistant Bilateral Upper and Lower Eyelid Ectropion Postburn Injury

**Published:** 2012-03-23

**Authors:** Lara Wetton, Aruna Wijewardena, Michael Miroshnik, John Vandervord

**Affiliations:** Severe Burn Injury Unit, Royal North Shore Hospital, Pacific Highway, St Leonards 2065, New South Wales, Australia

## Abstract

**Objective:** The aim of this surgical procedure was to definitively correct severe recurrent upper and lower bilateral eyelid ectropion after 2 attempts with full-thickness skin grafts. It was necessary to repair all 4 eyelids with forehead skin. Middle lamella support was required. **Methods:** Bilateral median forehead flaps, which were well vascularized by the supratrochlear vessels, were chosen for this procedure to utilize the readily available forehead tissue. The median forehead flap can be easily positioned to cover the entire eyelid. Furthermore, the flap could be split in half, without the risk of vascular compromise. Middle lamella support was provided with a cartilage graft from the nasal septum. **Results:** At 4 months, the patient no longer suffered from exposure keratopathy and both forehead flaps had healed well. At 12 months, the functional and cosmetic outcomes of this procedure were acceptable. **Conclusions:** This method of surgery can be effective in the young, in whom the Mustarde cheek advancement fails, or when there is little available unburnt tissue. It can be used as an alternative to a forehead flap when both upper and lower eyelids are damaged bilaterally. The split forehead flap definitively corrected the ectropion in this case.

## CASE REPORT

The patient was a 38-year-old man, who was admitted to the Royal North Shore Hospital on September 20, 2008, transferred from Port Macquarie Hospital. His injury was caused by a petrol drum igniting and exploding at work. He sustained 40% total body surface area full-thickness and partial-thickness flame burns to his face and body. On the day of admission, he had a full-body debridement and his face was dermabraided. His intensive care unit stay was complicated by the development of renal failure. At approximately 2 months, a second debridement was performed and a split skin graft was applied to his lower face.

**Figure 1:**
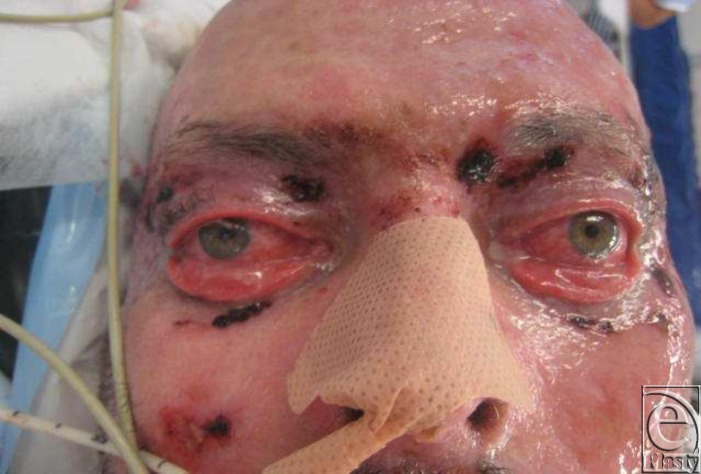
Six weeks postinjury. Photograph taken prior to the application of full-thickness skin graft to upper and lower lids bilaterally.

Because of his poor medical condition, his eyelids were addressed at 6 weeks (Fig 1). He steadily developed bilateral resistant ectropion over the ensuing 2 months despite 2 attempts at correction with full-thickness skin grafts (FTSG).

A split forehead flap was used to definitively correct his ectropion. This was chosen for several reasons: there was a lack of viable tissue in the lower/two thirds of the face; there was readily available forehead tissue; and forehead flaps were well vascularized and could be easily positioned to cover the entire eyelid. Septal cartilage could be used for middle lamella support. A free flap was considered, but most of his body, including both forearms, was burnt, so there was little possibility of accessing enough unburnt tissue.

**Figure 2:**
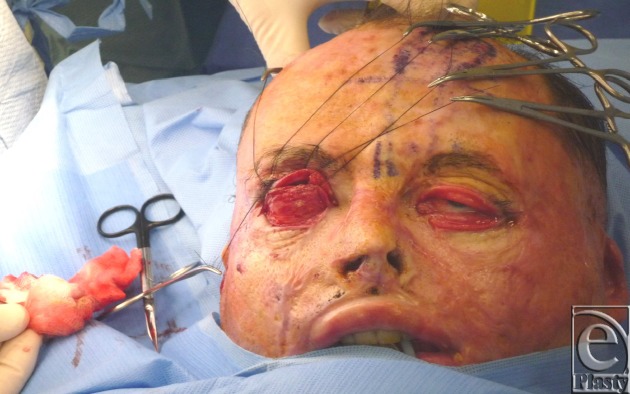
Exposure of the right orbital rim.

First, the defects were re-created in the area of the FTSG on the right eye, and the right lower orbital rim was exposed to the arcus marginalis (Fig 2).

Middle lamella support was provided by septal cartilage, which was harvested by an intranasal approach. It was inset into the right orbital rim after scoring to allow for cartilage to follow the curvature of the orbital rim.

**Figure 3:**
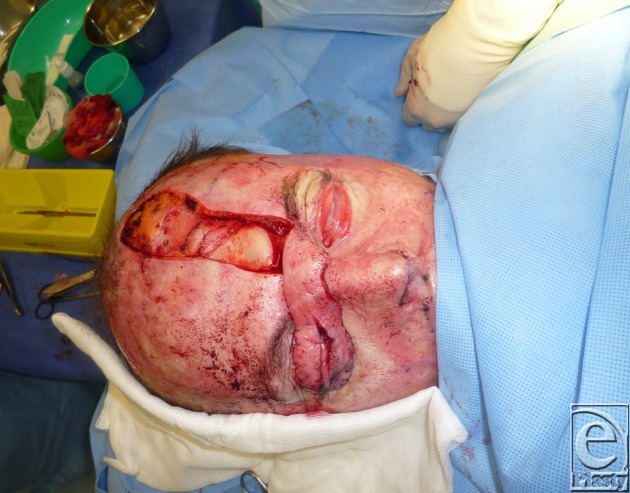
Flap split longitudinally.

A left paramedian forehead flap was raised on left supratrochlear vessels. The flap was thinned, split in half, and inset into the upper and lower eyelid defects (Fig 3).

After mid forehead, the supratrochlear vessel divides into multiple branches. The flap could be split in half, without the risk of vascular compromise.

The donor region from the left forehead was closed with an FTSG from the groin. After 10 days, the functional result was excellent and at 14 days, the flap was divided.

**Figure 4:**
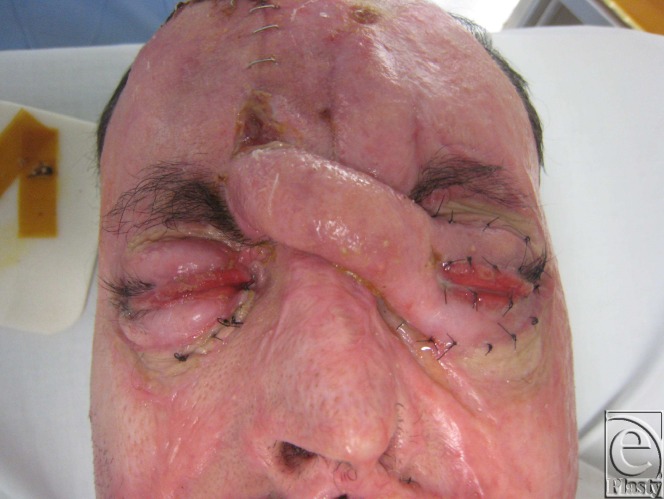
Predivision of the right forehead flap applied to the left eye.

Approximately 5 weeks later, a similar procedure was done for the left eye (Fig 4). Again, the flap was divided at 2 weeks.

**Figure 5:**
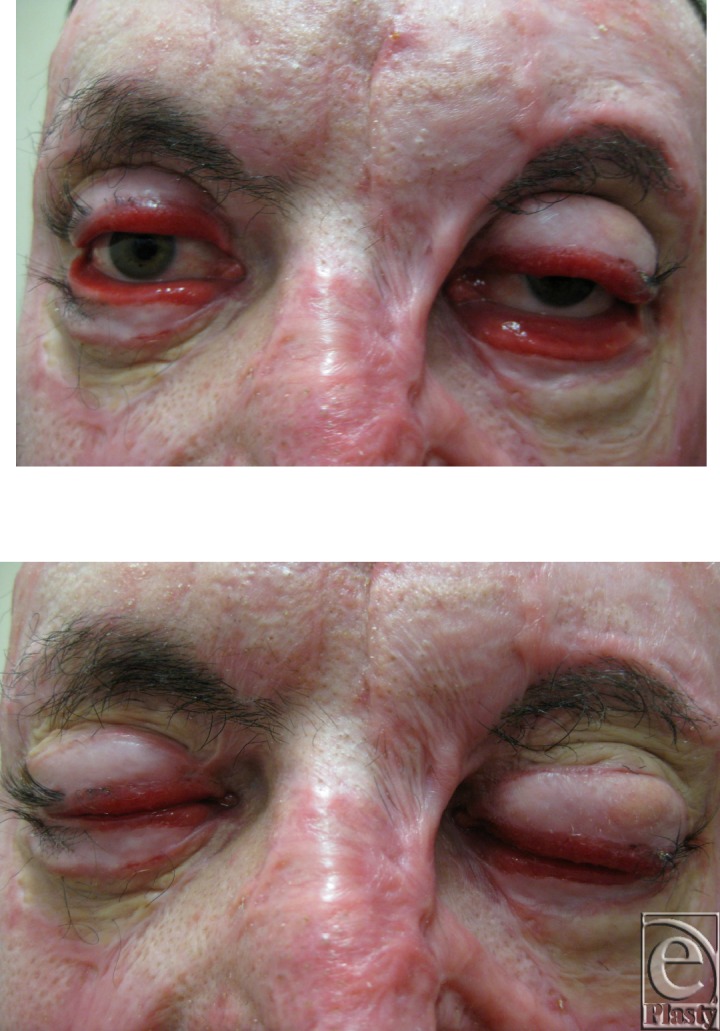
At 12 months.

At 4 months, the patient no longer suffered from ectropion and had good eyelid closure. Although good functional outcomes were the main aim of this procedure, Mr PK was happy with the cosmetic result. A photograph taken at 1 year demonstrates good eyelid closure and adequate globe coverage (Fig 5).

## DISCUSSION

Full-thickness burns to the face can result in varying degrees of scaring around the eyes, causing eyelid damage and ectropion. Ectropion describes the clinical situation in which the edge of the eyelid falls away from the globe and no longer protects the globe. In this setting, drainage of tears is impaired and the eyelid does not close properly, exposing the cornea to the outside environment. As a result, the patient can suffer from exposure keratopathy, corneal ulceration, and blindness. Moreover, visual acuity is poor, because the blinking action of the lid is impaired, and so the lid does not sweep the tears evenly across the globe.^1^

There have been various reconstructive procedures, including FTSG, local flaps, regional flaps, and free flaps, described in the literature to treat postburn eyelid ectropion. It is extremely rare for a bilateral upper and lower lid reconstruction to be performed postburn injury. Most procedures used in the past have been applied only to 1 eyelid, or 1 eye, unlike this case, in which 4 eyelids needed to be repaired.

The aim of surgical intervention, such as that of other contractures, is to release the thickened tissue, re-create the defect, and replace the tissue shortage with similar viable tissue.

Full-thickness grafts have been used in the past and remain the mainstay of treatment of uncomplicated cases, yielding good results. They are preferred to split skin grafts because they contract less.^2^^,^^3^ Early grafting with FTSG (within 7 days) prevents the recurrence of eyelid ectropion postburn injury.^4^ However, the lateral/one third of the lower eyelid is especially prone to the redevelopment of ectropion,^5^ particularly in the aged.

Regional flaps from the upper lid to the lower lid with a medially based pedicle have been used effectively for small defects, but when surrounding facial tissue is damaged, this becomes challenging.^1^ In Turkey in 1999, a laterally based orbicularis occuli myocutaneous flap from the upper lid was used to reconstruct a defect in the lower lid postburn injury for ectropion with a satisfactory outcome.^5^ Midline glabellar and median forehead flaps have also been used for reconstruction of the medial canthal area with pleasing results.^1^ In the case of Mr PK, both upper and lower lids needed to be repaired bilaterally, so a transposition flap from the upper to the lower lid was not an option.

Local flaps, such as the cheek advancement flap introduced by Mustarde, have a place especially in older patients in whom the skin is hypoelastic. This does, however, rely on the presence of surrounding unburnt tissues. A mucosal membrane graft from the hard palate has been used in conjunction with this flap to replace the damaged conjunctivae.^6^ A supraorbital Fricke flap has been attempted, but this results in an updrawn brow^7^ and a poor aesthetic outcome. Mr PK's face was badly scarred, so a Mustarde flap, or another regional flap, was not an option.

Various free flaps have been used to correct ectropion postburn injury and could have been considered for our patient. Two cases using a radial forearm flap have been described; one^8^ in 1995 and another^9^ in 1997. In 1999, a patient sustained a severe facial burn in which there were no facial donor sites and a dorsalis pedis free flap was used to cover the exposed cornea after bilateral conjunctival advancement flaps were used with septal cartilage graft for support.^10^ More recently, a 2008 case report describes an FTSG failure for ectropion, and finally definitive treatment with an anterolateral thigh flap.^11^ In our case, Mr PK had 40% mixed-thickness burns on his body and both arms were severely burnt, so the use of a free flap was considered but not attempted.

Lamella support with a cartilage graft has been used for many years by reconstructive surgeons. More recently, acellualar human dermal allograft as a tarsus substitute after chemical and thermal burns was used in a study of 13 patients with excellent results.^12^ A case in which a chondromucosal graft from the nasal septum (used as the deep layer of the upper eyelid) was used to correct ectropion post–electrical burn, and that provided adequate support to the lid, has also been described.^13^ Autogenous tarsal grafts have been used to correct lower eyelid ectropion from a variety of causes and improve the functional and cosmetic outcome of the eyelid.^14^ In the case of Mr PK, previous FTSG failures and irreparable damage to the middle lamella meant that support was needed for a pillar in which to place a flap onto. Septal cartilage, which was accessible and available, provided the support needed for the lamella.

Mr PK had forehead skin, which was of an appropriate caliber to be used as a flap. He had partial-thickness burns to his forehead skin. Full-thickness burns to the forehead would make it difficult to undertake this procedure, because of severe scarring, contraction, and damage to the supratrochlear vessels. A procedure was needed which would repair all 4 eyelids with forehead skin. This would not be easy with a simple median forehead flap. The forehead flap required adequate blood supply, which was provided by the supratrochlear vessels. This method of surgery can be effective in the young, in whom the Mustarde cheek advancement fails, and as an alternative to a forehead flap when both upper and lower eyelids are damaged bilaterally. Modifications, such as timing, may be considered, especially if the flap must be raised on previously burnt tissue. This forehead flap reconstruction was performed at 6 months postburn injury. Forehead skin, if burnt at all, should have time to heal almost completely before undertaking this procedure.

Innovative techniques such as these have been employed only as salvage when FTSG have failed. Perhaps, there is a group of patients with severe burn injury who would benefit from this type of surgery at the outset. However, this subset of patients would be difficult to define and a set of clinical signs and symptoms would be needed to grade the severity of ectropion.

The split forehead flap definitively corrected the ectropion with a pleasing functional and aesthetic outcome.
